# Drug-Induced Hepatitis in Children: The Experience of a Single Center in Romania

**DOI:** 10.3390/children9081136

**Published:** 2022-07-29

**Authors:** Irina Dijmărescu, Oana Maria Guță, Livia Elena Brezeanu, Adrian Dumitru Dijmărescu, Cristina Adriana Becheanu, Daniela Păcurar

**Affiliations:** 1 Department of Pediatrics, “Grigore Alexandrecu” Emergency Children’s Hospital, 011743 Bucharest, Romania; oana-maria.guta@umfcd.ro (O.M.G.); livia-elena.brezeanu@rez-umfcd.ro (L.E.B.); daniela.pacurar@umfcd.ro (D.P.); 2 Department of Pediatrics, “Carol Davila” University of Medicine and Pharmacy, 020021 Bucharest, Romania; 3 Radiology Department, Fundeni Clinical Institute, 022328 Bucharest, Romania; adrian.dijmarescu@umfcd.ro

**Keywords:** acute hepatitis, drug-induced liver injury, paracetamol, albendazole

## Abstract

Drug-induced liver injury (DILI) is uncommon but potentially lethal. Over 6 years, 2533 children with acute liver disease were identified in our center, 48 of which suffered from toxic hepatitis, and 40 exhibited DILI (22 paracetamol-related, 14 albendazole-related). The most affected children were in the 13–17-year-old age group. The mean time between drug ingestion and disease diagnosis was 25.4 h for paracetamol-related DILI and 21.6 days for the albendazole-related group. Clinical features were mostly gastrointestinal, jaundice being reported in 30% of the cases. Regarding the type of liver injury, for 70% of the patients it was hepatocellular (mostly paracetamol toxicity), for 11% cholestatic, and for 19% mixed (albendazole-related). The mean initial ALT value was 1020 U/L for all DILIs. Coagulopathy was only identified for the acetaminophen-related group. The median number of hospitalization days was 6.9 for DILI related to acetaminophen ingestion, compared with 7 for the idiosyncratic pattern. When applying the DILI severity index, 81% of the patients were categorized as having a mild hepatic ailment, while 19% had a moderate–severe or severe disease. No deaths were reported in the study group. The diagnosis of DILI involves the exclusion of other causes of liver injury; therefore, it is considered one of the most challenging diagnoses in hepatology.

## 1. Introduction

Drug-induced liver injury (DILI) is one of the most challenging liver disorders faced by pediatric hepatologists [1]. DILI is an uncommon but potentially lethal adverse drug reaction. In population-based studies using different methodologies and cut-offs, the crude annual incidence of DILI post-marketing ranged from 2.4 to 13.9 per 100,000 inhabitants [2].

DILI consists of altered liver function as a consequence of exposure to hepatotoxic substances or their metabolites. In 2011, an international expert working group meeting proposed a new definition for DILI based on the following criteria: (1) alanine aminotransferase (ALT) elevation ≥5 upper limit of normal (ULN); (2) alkaline phosphatase (ALP) elevation ≥2 ULN in the absence of known bone pathology leading to increases in the ALP level (particularly, with accompanying elevations in gamma-glutamyltransferase (GGT) concentrations); or (3) ALT ≥3 ULN and simultaneous elevation of total bilirubin concentrations above 2 ULN [3,4].

Pharmacologically, there are two types of DILI: dose-dependent or intrinsic and dose-independent or idiosyncratic. Dose-dependent DILI (such as that due to acetaminophen overdose) appears when the drug dose exceeds its known limit for toxicity; it is predictable and reproducible, there is a short period of latency between exposure and DILI manifestations, and liver damage is proportional to the administered dose. Idiosyncratic DILI (such as that due to albendazole exposure) is unpredictable, the period of latency is variable, and liver damage is not dose-dependent, frequently developing at therapeutic doses [1,2].

Acetaminophen is one of the most commonly used drugs in pediatrics, due to its analgesic, antipyretic, and anti-inflammatory effects. When used in therapeutic doses, paracetamol has few side effects. It is metabolized by phase II conjugating enzymes, mainly UDP-glucuronosyltransferase (UGT) and sulfotransferase (SULT), converting the drug to nontoxic compounds, which are subsequently excreted in the urine. Approximately 5–9% of the drug’s metabolism is achieved through cytochrome P450 enzymes (CYP), especially CYP 2E1, resulting in a highly reactive intermediate metabolite, N-acetyl-p-benzoquinone imine (NAPQI), which is detoxified by conjugation with glutathione (GSH). Only 2% of acetaminophen is excreted unchanged in the urine [5]. Acetaminophen overdose leads to the saturation of phase II metabolizing enzymes, excessive NAPQI, and the depletion of GSH, causing a covalent binding of sulfhydryl groups in mitochondrial proteins [5]. The result is endoplasmic reticulum (ER) stress, mitochondrial oxidative stress and dysfunction, the presence of superoxide radicals and hydrogen peroxide (H2O2), and Krebs cycle and β-oxidation dysfunction with ATP depletion and the opening of mitochondrial permeability transition pores, with the translocation of mitochondrial proteins. This results in nuclear DNA fragmentation, necrosis of the hepatocytes, and, thus, acute liver injury, which may become life-threatening [5,6].

Albendazole is a benzimidazole derivative with a wide anthelmintic spectrum, acting through the metabolite albendazole sulfoxide, synthetized after the first hepatic pass. Albendazole sulfoxide is mainly bound to plasma proteins (up to 70%) and is distributed throughout various tissues. The excretion of albendazole sulfoxide is probably most common through the biliary system [7].

There is limited information about the immune responses induced by drugs, including drugs which are metabolically activated, but cluster of differentiation (CD) 86 and CD54 expression levels on monocytic cells were notably upregulated by treatment with albendazole in the presence of CYP3A4 [8].

Patterns of liver injury—hepatocellular, cholestatic, or mixed—have been defined biochemically by the ALT and ALP levels when compared with the reference levels and/or the R ratios between actual ALT/ALT(ULN) and actual ALP/ALP (ULN) at presentation [9].

The widely used scores for causality assessment of DILI, the Roussel Uclaf Causality Assessment Method (RUCAM) score and the Maria and Vittorino score, are used as suggestive scores for idiosyncratic DILI [2,10]. The Drug-Induced Liver Injury Network (DILIN) developed a 5-point scale for grading the severity of liver injury (DILI severity index) based upon the presence of jaundice, hospitalization, signs of hepatic or other organ failure, and final outcome [11,12].

DILI is mostly identified based on a diagnosis of exclusion; therefore, detailed history and anamnestic data, such as the time of exposure, period of latency, and effect of a subsequent exposure, must be carefully assessed. In the absence of diagnostic tests and/or biomarkers, the diagnosis of DILI requires a high index of suspicion after diligently excluding other causes of abnormal liver tests [1].

In this paper, we report a cohort of 40 children with DILI, enrolled in a single pediatric tertiary center. The research focused on the most common hepatotoxic agents (acetaminophen and albendazole), the epidemiological and clinical presentation and laboratory findings of the patients, and severity scores of the cohort studied. The rationale behind writing this report was the ease in prescribing this medication by physicians and the high accessibility of some toxic medications to children, but also the low level of physician awareness of the importance of reporting DILI cases. The aim of this study was to present an update on the diagnostic approach to each type of DILI, DILI natural history, and the current management strategy, based on clinical and biochemical findings, to improve the diagnostic accuracy and achieve a faster diagnosis of DILI.

## 2. Materials and Methods

We conducted a retrospective and prospective observational study that included pediatric patients aged 1 to 17 years with acute liver disease, admitted to the Pediatrics Department of “Grigore Alexandrescu” Emergency Children’s Hospital in Bucharest over a period of 6 years. We collected data on the patients’ age, possible causes of liver injury, type of substance administrated and its potential role on DILI, associated symptoms, laboratory assessments, outcomes, etc.

After informed consent was obtained, an extensive medical history was recorded, including recent alcohol or medication consumption, smoking history, drug intolerances, and symptoms in the 3 months leading to DILI onset. A complete physical examination was performed, and detailed information regarding all relevant laboratory and imaging studies was reviewed to ensure that the patients met the enrolment criteria. For all patients, the abnormal liver tests were repeated within 24 h for confirmation.

Using the eligibility criteria for acute liver disease, we enrolled patients with ALT elevation ≥5 ULN or ALP elevation ≥2 ULN in the absence of a known bone disorder justifying the increase in ALP level (with elevations in GGT concentrations), or ALT ≥3 ULN and simultaneous elevation of total bilirubin concentration >2 ULN, or an INR above 1.5 in the absence of another cause of hyperbilirubinemia or hypoprothrombinemia.

The patients were labelled as DILI patients after excluding infectious diseases, autoimmune liver disease, metabolic disorders, diseases of the biliary tract, and non-alcoholic steatohepatitis. The laboratory tests also aimed to assess the severity of liver involvement. The following investigations and assays were performed:

- Screening tests for hepatitis A, B, C, D, E, Epstein–Barr virus, herpes virus, adenovirus, cytomegalovirus, parvovirus B19, and SARS-CoV-2 (if appropriate);
- Assessment of the erythrocyte sedimentation rate, albumin levels, gamma globulins, immunoglobulin G, antinuclear antibodies (ANA), smooth muscle antibodies (SMA), and liver–kidney microsome antibodies (LKM1);
- Alpha 1 antitrypsin serum level;
- Serum ceruloplasmin level, 24 hour urine copper;
- Sweat test;
- Immunoglobulin A and immunoglobulin G tissue transglutaminase antibodies;
- Thyroid hormones levels
- Total lipids, triglycerides, and cholesterol levels and coagulation profile;
- Abdominal ultrasound.

Data analysis led to defining the type of liver lesion and the pharmacological mechanism and to determining the scores for severity and causality. Microsoft Excel 2010 was used for organizing and analyzing the data.

## 3. Results

**Figure 1:**
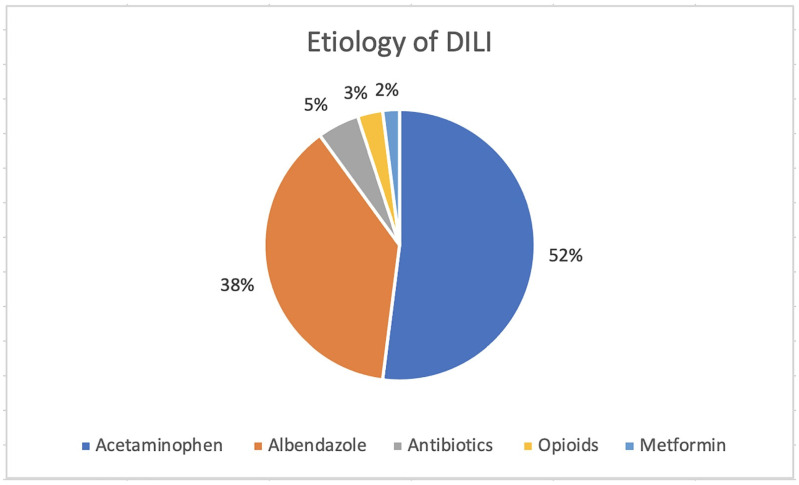
Etiology of DILI.

We identified 2533 children aged 1 to 17 years with suspected acute liver injury. Out of a total of 2533 hospitalized children for hepatic disease, 48 (1.89%) patients were found to have toxic hepatitis; among them, only 40 patients were diagnosed with DILI according to the definition. A total of 22 patients with paracetamol-related DILI and 14 children with albendazole-related DILI were admitted to our center during the analyzed period of time (representing 90% of the total of DILI patients). There were only three other classes of medication involved in DILI in our cohort—metformin (one patient), opioids (one patient), and antimicrobials (one case of DILI after 17 days of ampicillin/sulbactam treatment and one case after isoniazid overdose for suicidal purposes in a patient who was being treated for tuberculosis). Other common substances involved in hepatotoxicity were insecticides, heavy metals, and mushrooms (Figure 1).

The mean dose for acetaminophen was 128 mg/kg (ranging between 98 and 221 mg/kg). Albendazole was administrated without medical recommendation to eight patients, and for suspected giardiasis or oxyuriasis in the other cases.

**Figure 2:**
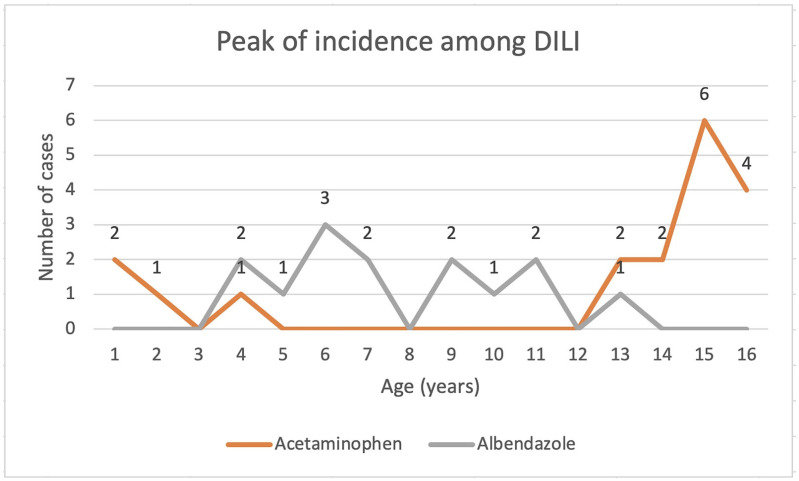
Peak of incidence among DILI patients.

From the entire cohort of DILI, the most affected children were in the 13 to 17 years age group (52.5%), with a peak incidence in adolescents aged 15–16 years (27.5%). The incidence curves based on age for DILI secondary to acetaminophen and albendazole are presented in Figure 2.

For the paracetamol overdose DILI group (intrinsic DILI), the time between the ingestion and the identification of liver cytolysis, at the time of medical evaluation, ranged from 6 to 49 h (mean 25.4 h), whereas for the albendazole group (idiosyncratic DILI), the time from ingestion to symptom onset or the identification of liver injury ranged between 3 and 89 days (mean 21.6 days).

Among the symptomatic children, the most common clinical features were abdominal pain (50%), followed by nausea and vomiting (45%); in most cases, these symptoms were reported in the acetaminophen-related DILI group. Jaundice was only observed in 30% of cases, of which nine were in the albendazole-related DILI group, and only three were in the acetaminophen overdose DILI group. Four patients complained of a headache, and three others reported fever, symptoms suggestive of an associated acute illness. No patients were identified to have renal or neurologic complications in our cohort. None of the children had a known history of pre-existing liver disease. Six children (15%) were asymptomatic, the diagnosis of DILI being established after incidentally identifying liver cytolysis and excluding other liver diseases.

**Figure 3:**
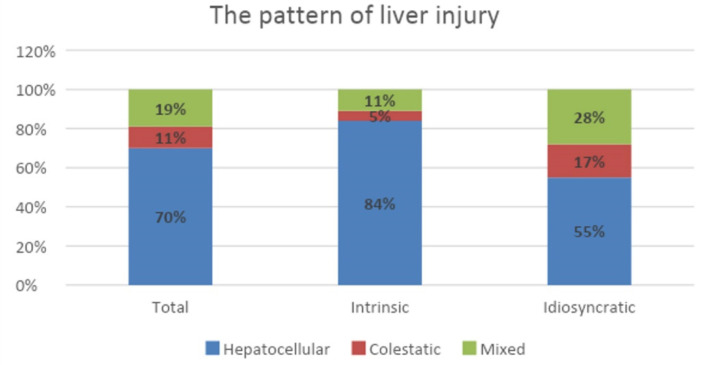
Patterns of liver injury.

In our cohort, according to the R ratio, we noticed hepatocellular injury in 70% of cases, cholestatic injury in 11%, and mixed injury in 19% (Figure 3).

The types of liver injury, determined based on the R ratio for the paracetamol and the albendazole groups, were different: we observed that, for intrinsic DILI (secondary to paracetamol overdose), the hepatocellular pattern was predominant (84% of cases), whereas for idiosyncratic DILI (as a result of albendazole ingestion), the cholestatic pattern and mixed injury were more common, at 17% and 28%, respectively.

The mean initial ALT value was 1020 U/L for all DILI cases (ranging between 18 and 8036 UI/L). The mean value of transaminases for acetaminophen-induced DILI patients was 1334 UI/L, whereas for the albendazole-induced DILI group, it was 714 UI/L.

The mean initial total bilirubin level in the DILI acetaminophen-related patient group was 1.28 mg/dL (ranging between 0.3 and 4.2 mg/dL), whereas for the conjugated bilirubin value, it was 0.38 mg/dL (ranging between 1.17 and 0.1 mg/dL). For the idiosyncratic DILI patient group, the total bilirubin mean value was 2.68 mg/dL (ranging between 0.43 and 17 mg/dL), and the conjugated bilirubin mean value was 1.81 mg/dL (ranging between 0.28 and 13.4 mg/dL).

The intensity of cholestatic syndrome should be indicated by ALP, whose mean value was normal; however, even if GGT is not a constant marker of cholestasis, its value was elevated, with a mean of 44.3 UI/L (ranging between 12 and 232 UI/L) for intrinsic DILI secondary to paracetamol overdose, compared with 80.2 UI/L (ranging between 11 and 239 UI/L) for idiosyncratic DILI. The idiosyncratic DILI pattern is mainly characterized by cholestatic syndrome with less intense cytolysis, whereas the intrinsic pattern presents more severe cytolysis with less intense cholestasis.

Coagulopathy, defined by an INR > 1.5, was identified in 12 cases (30%), whereas prothrombin activity was <80% for 16 patients (40%), all belonging to the acetaminophen-related DILI group.

The median number of hospitalization days was 6.9 (ranging between 1 and 54) for DILI related to acetaminophen ingestion, compared with 7 (ranging between 1 and 19 days) for idiosyncratic-pattern DILI.

When applying the DILI severity index (taking into consideration ALT, ALP, bilirubin, INR, hospitalization, and decompensated liver disease) [12], it is highlighted that 81% of the patients (29 patients) were categorized as having a mild hepatic disease, whereas 19% of them (7 patients) were classified as having a moderate–severe or severe disease. No deaths were reported in the study group. 

When applying the RUCAM score for the albendazole-related group in our research, its values confirm the high likelihood of liver disorder associated with medicine ingestion, the score ranging from 10 to 13 points for all patients. In one patient, re-exposure occurred.

The demographics, clinical parameters, and outcomes of the three DILI groups (acetaminophen, albendazole, and others) are presented in Table 1.

## 4. Discussion

According to the American College of Gastroenterology guidelines, hepatocellular liver injuries secondary to viral hepatitis (hepatitis A, B, C, and E, cytomegalovirus, Epstein–Barr virus, and herpes simplex virus infection), autoimmune hepatitis, vascular liver disease (Budd–Chiari syndrome and ischemic liver injury), and Wilson’s disease need to be excluded when considering DILI. For the cholestatic pattern, biliary obstruction and biliary autoimmune diseases, such as primary biliary cholangitis (PBC) and primary sclerosing cholangitis (PSC), were excluded. Other diagnoses that should be excluded are hemochromatosis, alfa1-antitrypsin deficiency, non-alcoholic fatty liver disease (NAFLD), and non-alcoholic steatohepatitis (NASH) [13].

Acute hepatotoxicity in children may be the result of an intentional or unintentional overdose. The latter could be the consequence of accidental ingestion, frequent administration, the use of associated hepatotoxic drugs, or the use of multiple acetaminophen-containing products [14,15].

More than one-half of our patients were admitted due to acetaminophen-induced liver injuries, which is in line with data from the United States, where acetaminophen is reported to be the most common drug involved in DILI-associated acute liver failure. Acetaminophen is a standard choice for contemporary pain management and antipyretic treatment in a variety of patients, including children. Some studies reported acetaminophen to be safe for use in preterms. The pediatric posology is 10 to 15 mg/kg/dose every 4 to 6 h, not exceeding five doses in 24 h, the maximum daily dose being 75 mg/kg/day. Usually, these doses are considered risk-free, but hepatotoxicity has been reported even with doses under the recommended limits. Toxicity is likely to occur at doses >150 mg/kg [16]. In our cohort, acetaminophen overdose occurred secondary to accidental ingestion in unsupervised children who were already receiving this medication or as voluntary ingestion in adolescents with suicide attempts. The mean dose of acetaminophen that caused suggestive symptoms and liver cytolysis was 128 mg/kg (ranging between 98 and 221 mg/kg), indicating metabolic particularities in children or a higher risk because of infections or substance associations (such as with alcohol and antibiotics). Recent clinical studies have validated the efficacy of new biomarkers for DILI prognosis, such as cytokeratin-18, macrophage colony-stimulating factor receptor, and osteopontin [17]. According to studies from other countries, acetaminophen is one of the most common drugs used with self-harm aims, particularly among young females [15].

The mechanism of injury is dose-related. Even if children are less susceptible to complications, due to the treatment with N-acetylcysteine, there are many cases of acute hepatic failure attributed to acetaminophen [14,16]. Self-intoxication is frequently seen among older children and adolescents.

In our research, in both the albendazole- and the paracetamol-related DILI groups, most children were adolescents, with a mean age of 7.42 and a median age of 12.91 years old. For the acetaminophen-related DILI group, most patients were aged over 12 years, and the medication was ingested voluntarily, in the absence of any medical recommendation. Almost all children in the albendazole cohort were aged under 12 years; the medication was administered according to dosage, but not always based on medical recommendations. Our study demonstrated an increased incidence among adolescent girls in the group of patients with DILI acetaminophen overdose, the ingestion being intentional, for suicidal purpose; only for one patient was the ingestion unintentional (accidental), raising the issue of possibly negligent parental care.

The onset of acute intoxication with acetaminophen is rapid. Usually, it starts with the marked elevation of ALT and aspartate aminotransferase (AST) serum levels in 24 to 72 h after ingestion, followed by clinical symptoms in the following 48 to 96 h. The first stage is also called asymptomatic or with minimal clinical changes. Right upper quadrant pain, elevation in liver enzymes, alteration of coagulation parameters, and in severe cases, evidence of nephrotoxicity or pancreatitis, may appear after 24 h. Evidence of liver failure and multi-organ failure is common 72 h after ingestion, and in this stage, death might also occur. When attained, recovery is seen in 4 to 14 days [6]. Gastrointestinal disturbances are described in the literature as associated with many diseases in the pediatric field, including infections and poisoning, but they are also the most frequent symptoms of DILI, a fact confirmed by our study [6,18].

In our research, among the symptomatic children, the most common clinical features were abdominal pain (50%), followed by nausea and vomiting (45%); in most cases, these symptoms were reported in the acetaminophen-related DILI group. Liver injury secondary to acetaminophen toxicity was indicated by high levels of liver enzymes (above 10 ULN), with no cholestasis but with coagulopathy in 36% of children.

DILIN research which included 30 children with idiosyncratic DILI demonstrated that more than one-half of the cases were caused by antimicrobial medication and tuberculostatic agents, followed by anticonvulsant drugs, with albendazole-related liver disease not being mentioned [19]. Our research, on a similar number of pediatric patients, over the same period of time, reports albendazole as the main cause of idiosyncratic DILI.

It is known that in cases of albendazole administration, the elevation of liver enzymes is often observed, with return to normal values after drug discontinuation, without any further complications. Few cases of obstructive jaundice have been reported [20]. Rare cases of acute liver failure and fulminant hepatitis have also been described [21]. The mechanism by which albendazole affects the liver function and causes DILI is not well established. It is considered to be an idiosyncratic reaction involving immunological processes [20]. In pediatric patients, the posology is 15 mg/kg/day, up to a maximum daily dose of 800 mg [22], and adverse reactions usually occur with prolonged therapy (>7 days of treatment) and high doses of medication and are mostly mild and transient. In our cohort, patients who developed DILI secondary to albendazole ingestion had received the correct medication dose for an appropriate time with one exception, where the patient was administered the medication for 10 days. Hence, the individual immunological specifics are probably responsible for the development of the liver ailment. Liver disease secondary to albendazole administration has been reported when using a maximum of two courses (repeated at 2 or 3 weeks) and therapeutic doses over 1 to 5 days, in patients with no prior liver impairment. Liver toxicity was less pronounced (28% of the patients were even asymptomatic) in the presence of an important degree of cytolysis and cholestasis and lower than in the case of dose-related DILI. Coagulopathy was identified in 21% of these patients.

Hepatocellular DILI is characterized by ALT ≥ 5 times greater than the ULN for an ALT/ALP ratio ≥5 times greater than the ULN. Cholestatic DILI is defined by ALP ≥2 times greater than the ULN or an ALT/ALP ratio ≤2 times greater than the ULN; mixed DILI is diagnosed when the ALT/ALP ratio is <5 but >2 times greater than the ULN [4,6].

In our cohort, according to the R ratio, we noticed hepatocellular injury in 70% of the cases, cholestatic injury in 11%, and mixed injury in 19%. We found previous research reporting that hepatocellular injury patterns are more common in younger patients, whereas cholestasis prevails in older patients [23]. These findings are compatible with those of our study.

Scores that use more parameters are more appropriate for appreciating the actual disease severity compared to scores obtained using the ALT value alone.

The RUCAM score is a structured, standardized, and validated tool for assessing hepatotoxicity. It is mainly used for idiosyncratic reactions. Key items for RUCAM include clinical, biochemical, and serologic features [9,24]. When applying the RUCAM score for the albendazole-related group in our research, its values confirmed the high likelihood of liver disorder associated with medicine ingestion, the score ranging from 10 to 13 points for all patients.

When applying the DILI severity index (ALT, ALP, bilirubin, INR, hospitalization, and decompensated liver disease), 81% of the patients (29 patients) were categorized as having a mild hepatic impairment, whereas 19% (7 patients) were classified as having a moderate–severe or severe disease. No deaths were reported in the study group [9,12].

In order to diagnose DILI, the exclusion of other diagnoses is of utmost importance; therefore, providing accurate medical information to the patient and family is imperative, helping them better understand their condition, treatment options, and risks [25]. 

Limitations of our study include the retrospective design, lack of the date of disease onset (especially in cases with problematic parental cooperation), and no long-term follow-up of the patients.

## 5. Conclusions

The diagnosis of DILI involves excluding other causes of liver injury, so it is considered to be one of the most challenging diagnoses in hepatology. Here, the conditions of many patients diagnosed with toxic liver disease were secondary to paracetamol and albendazole use. DILI was most frequent among adolescent girls. Abdominal pain was one of the most significant clinical features. For intrinsic DILI, the predominant pattern was hepatocellular injury, whereas for idiosyncratic DILI, cholestatic syndrome was predominant. Our study contributes to the understanding of drug-induced liver injury, even if our data may not be applied to all DILI patients.

## Figures and Tables

**Table 1:** Demographics, clinical parameters, and outcomes of patients with DILI.

Characteristics of DILI Patients			
	Albendazole	Acetaminophen	Others
Number of cases	14 (38%)	22 (52%)	4 (10%)
Age (years)	7.71 (4–13)	12.91 (1–17)	9 (1–15)
Time from exposure	21.6 (3–89) days	25.4 (6–49) h	8.6 days (8 h–34 days)
Clinical examination			
Abdominal pain	4 (28.6%)	16 (72.7%)	0
Vomiting	3 (21.4%)	14 (63.6%)	1 (25%)
Jaundice	9 (64.3%)	3 (13.6%)	0
Fever	0	1 (4.5%)	2 (50%)
Headache	0	3 (13.6%)	1 (25%)
Asymptomatic	4 (28.6%)	1 (4.5%)	1 (25%)
laboratory tests			
ALT (UI/L)	714 (12–2070)	1334 (13–8036)	369 (16–1148)
AST (UI/L)	390 (30–1252)	1128 (12–9660)	339 (32–526)
ALP (UI/L)	111 (79–186)	56.6 (18–280)	180.7 (108–262)
GGT (UI/L)	80.2 (12–239)	44,3 (12–232)	64 (23–190)
Total bilirubin (mg/dl)	2.68 (0.43–17)	1.28 (0.3–4.2)	1.67 (0.7–3.1)
Conjugated bilirubin (mg/dL)	1.81 (0.28–13.4)	0.91 (0.3–3.03)	0.7 (0.3–2.2)
INR > 1.5	3 (21.4%)	8 (36.4%)	1 (25%)
DILI type			
Hepatocellular type	10 (55%)	16 (84%)	2 (50%)
Cholestatic type	3 (17%)	1 (5%)	1 (25%)
Mixed type	5 (28%)	2 (11%)	1 (25%)
Length of hospitalization	7 (1–19)	6.9 (1–54)	2.8 (1–7)

## Data Availability

Data are available on request.
